# Teaching LIVES as First Line Support for Women Affected by Violence Using Psychodrama Methods: An Erasmus+ Experience

**DOI:** 10.1192/j.eurpsy.2026.11960

**Published:** 2026-07-31

**Authors:** I. G. Yilmaz-Karaman, A. Ventriglio

**Affiliations:** 1 Psychiatry, Eskisehir Osmangazi University, Eskisehir, Türkiye; 2 Psychiatry, University of Foggia, Foggia, Italy

## Abstract

**Introduction:**

The World Health Organization (WHO) recommends the LIVES approach—Listen, Inquire, Validate, Enhance safety, and Support—as a cornerstone for providing first-line support to women affected by violence in healthcare settings. This model emphasizes empathetic listening, careful inquiry into needs, validation of women’s experiences, attention to safety, and the provision of appropriate support and resources. Implementing LIVES is essential, as healthcare professionals are often the first point of contact for women experiencing violence.

**Objectives:**

This study aimed to teach the LIVES approach to the psychiatry residents while adapting psychodrama techniques. The project was implemented within the scope of an Erasmus+ collaboration.

**Methods:**

During the exchange period, dedicated sessions were organized for two hours over three consecutive days, focusing on the concepts of sex and gender, gender-based discrimination, the definition of violence against women, and the WHO LIVES approach. Psychodrama techniques such as role-play, doubling, mirroring, and role reversal enabled participants to embody both healthcare providers and women affected by violence, fostering empathy and a deeper understanding of the LIVES model. Each session was followed by group sharing and reflective discussions to consolidate knowledge, connect experiences to real-world practice, and strengthen the sense of collective responsibility.

The number of participants varied from 9 to 14. Qualitative feedbacks were collected via Google Forms (n=4).

**Results:**

The participating psychiatry residents reported that the program increased their awareness of gender-based violence and improved their self-efficacy in delivering first-line support in line with the LIVES approach. They emphasized that the psychodrama-based experiential format fostered empathy, perspective-taking, and a deeper appreciation of women’s experiences of violence. Compared to conventional didactic methods, the interactive design was perceived as more effective in stimulating emotional engagement, critical reflection, and the ability to apply theoretical knowledge in clinical encounters.

**Conclusions:**

This Erasmus+ training demonstrated that integrating psychodrama techniques into teaching the WHO LIVES approach is a promising and innovative method for preparing healthcare professionals to respond effectively to women affected by violence. By combining experiential learning with reflective practice, the program not only enhanced knowledge and empathy but also strengthened participants’ confidence in applying first-line support skills in clinical contexts.

**Disclosure of Interest:**

None Declared

